# A Four-year Review of Delayed Initial Treatment of Patients with Congenital Talipes equinovarus in a General Hospital

**DOI:** 10.5704/MOJ.1503.007

**Published:** 2015-03

**Authors:** DI Yoyos

**Affiliations:** Department of Orthopaedics, Hasan Sadikin General Hospital, Bandung, Indonesia

**Keywords:** CTEV, delayed initial treatment, prevention of poor outcome

## Abstract

Congenital Talipes Equinovarus (CTEV), or club foot, is a common congenital orthopaedic abnormality of the foot, which is easily diagnosed but difficult to treat perfectly. Controversy in terms of its etiology, classification and management continues to exist. Delayed initial treatment in patients with clubfoot has a strong correlation with a poor outcome. The purpose of this study was to review the factors that influence the outcome in patients who get delayed initial treatment at Hasan Sadikin General Hospital so that poor outcome can be prevented. We reviewed the medical records of 15 patients (23 feet) during the period from January 2009 to December 2013 and analysed various factors including gender, age at time of first treatment, type of disorder, the level of success of non-surgical therapy, parent education level, family income and accessibility to health care centre. CTEV was more common in girls in our patients who were in the 6–12 months age group. The most common type of CTEV was the flexible type. Treatment with serial casting produced good results in most patients. The majority of parents' educational level was junior high school and had 2–5 million/month income. The accessibility of patients to health care centre was difficult.

## Introduction

Congenital Talipes Equinovarus (CTEV), or club foot, is a common congenital orthopaedic abnormality of the foot, which is easily diagnosed but difficult to treat perfectly. First described by Hippocrates, Controversy in terms of the aetiology, classification and management of club foot continues to exist^1,2,3,4^.

The incidence of CTEV is 0.93 to 1.5 per 1,000 births in the Western population, while in the Eastern region the incidence is 0.6 per 1,000 births. The condition was two times more common in the male child and 50% had bilateral involvement^2,5^. The “golden period” for commencement of treatment is three weeks after birth, since up to the age of less than three weeks, ligaments in the feet are still pliable so that they can be manipulated. Treatment is considered delayed after 6 months of age.

Typically in developing countries where clubfoot was often neglected, females were more likely to abuse. However, it is still questionable if the female gender of the affected child affects the motivation of the parents to look for an early treatment for the child. Age distribution of the patients would give an indication for the delayed treatment in the region.

Ponseti classifies the clubfoot as (1) typical or flexible and (2) atypical or rigid type, the latter being more difficult to treat.

In a developing country where parents' education level and income are low, most clubfoot treatment are generally delayed. This is further aggravated by the lack of health care services in the rural areas. These factors are probably relevant in a discussion of delayed initial treatment, and this study was undertaken to explore these factors so as to prevent poor outcome in future.

## Materials and Methods

This is a retrospective study of 15 patient (23 feets) of age more than 6 months at the time of initial presentation in our center, Hasan Sadikin General Hospital of Paediatric Division Orthopaedy & Traumatology outpatient clinic, during the period from January 2009 to December 2013. All patients with unilateral or bilateral CTEV were included in the study; excluded were those with other concomitant congenital abnormalities.

**Table I:** The Distribution Clubfoot By Age at First Presentation to Hospital

6–12 months	1–2 years	2–4 years	>4 years
11	3	1	0

**Table II:** The distribution of the Parent Education Level

Education Level	Frequency	%
Elementary School	2	13,3
Junior High School	7	46,6
High School	4	26,6
College	2	13,3

**Table III:** Distribution of Income Level of Parents

Income(IDR)	Frequency	%
< 2 million/month	4	26,6
2-5 million/month	8	53,33
> 5 million/month	3	20

## Results and Discussion

Age and sex: The clubfoot patients who received delayed treatment were mostly girls (9 patients out of 15) and most came to the healthcare service before the age of 1 year (Table I). It is surmised that the the female children were brought for treatment later in their life due to poor motivation of the parents to seek early treatment for the female child.

**Fig. 1:**
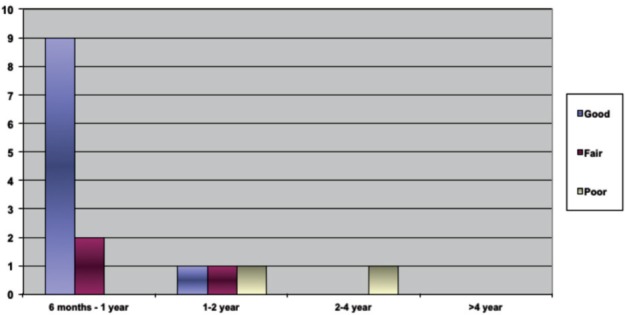
Results of treatment based on age when treatment commenced.

Type and management: Out of the 15 patients, 11 (73%), had flexible clubfoot and four (27%) had rigid clubfoot. Following treatment with the Ponseti method, ten patients had good result, three fair result, and 1 had a poor result (Fig. 1). Most fair and poor results were in patients who received initial treatment when they were older than one year of age.

Parents' educational level and income: Eight patient's parents only graduated from junior high school. Five parents graduated from high school, three from elementary school, and there were two who had graduated from College (Table II). Ten of the parents had 2-5 million/month income, five had less than 2 million/month income, and three parents had more than 5 million/month income (Table III).

These indicate a middle-low income level and probably quality of life in most patients in this study. Physical disabilities were generally not considered a real handicap in the society.

Health center accessibility: Nine parents experienced difficulty in getting to a health center while five others faced no difficulty. The difficulty in accessibility to a health care centre leading to poor health care of the people needs to be addressed by the relevant health authority.

## Conclusion

CTEV patients who received delayed initial treatment were more commonly female children and in the 6-12 months age group. The most common type of CTEV is the flexible type. Treatment with the Ponseti method is still recommended in clubfoot patients even with delayed initial treatment, in children below one year of age, as our results indicated. The majority of parents' educational level and income level is low The accessibility of patients to health care center is difficult.

Raising awareness and importance of early check in nearby health center must be promoted by relevant authorities, especially in people with low educational level and low income since clubfoot is more common in these sectors of the population.

The drawback of this study is the small number of patients. The conclusions drawn may be more relevant and valid in a larger cohort, with a longer follow-up of the patients treated to assess functional outcome and relapse.

Review of Delayed Initial Treatment of Patients with Congenital Talipes Eruinovarus in a District General Hospital
